# Association of single nucleotide polymorphisms in AXIN2, BMP4, and IRF6 with Non-Syndromic Cleft Lip with or without Cleft Palate in a sample of the southeast Iranian population

**DOI:** 10.1590/1678-7757-2017-0191

**Published:** 2017

**Authors:** Houshang Rafighdoost, Mohammad Hashemi, Hiva Danesh, Fatemeh Bizhani, Gholamreza Bahari, Mohsen Taheri

**Affiliations:** 1 Zahedan University of Medical Sciences, Cellular and Molecular Research Center, Zahedan, Iran; 2 Zahedan University of Medical Sciences, School of Medicine, Department of Anatomy, Zahedan, Iran; 3 Zahedan University of Medical Sciences, School of Medicine, Department of Clinical Biochemistry, Zahedan, Iran; 4 Genetic of Non-Communicable Disease Research Center, Zahedan University of Medical Sciences, Zahedan, Iran

**Keywords:** AXIN2, BMP4, IRF6, Non-syndromic cleft lip with or without cleft palate, NSCL/P, Polymorphism

## Abstract

Non-syndromic cleft lip with or without palate (NSCL/P) is a common congenital malformation worldwide, with complex etiology. It has been proposed that interaction of genes and environmental factors play a role in the predisposition to this disease.

**Objectives:**

The aim of this study was to examine the association between AXIN2 (axis inhibition protein 2) rs7224837, BMP4 (bone morphogenetic protein 4) rs17563, and IRF6 (interferon regulatory factor 6) rs861019 and 2235371 polymorphisms and NSCL/P in an Iranian population.

**Material and Methods:**

This case-control study was carried out on 132 unrelated NSCL/P patients and 156 healthy subjects. The variants were genotyped using polymerase chain reaction-restriction fragment length polymorphism (PCR-RFLP).

**Results:**

The findings suggest that BMP4 rs17563 polymorphism significantly decreased the risk of NSCL/P in codominant (OR=0.36, 95%CI=0.17-0.79, p=0.012, CT vs CC and OR=0.11, 95%CI=0.01-0.88, p = 0.019, TT vs CC), dominant (OR=0.30, 95%CI=0.15-0.62, p = 0.0007, CT+TT vs CC), recessive (OR=0.12, 95%CI=0.02-0.99, p = 0.023, TT vs CC+CT), overdominant (OR=0.39, 95%CI = 0.18-0.84, p=0.021, CT vs CC+TT), and allele (OR=0.28, 95%CI=0.15-0.55, p<0.0001, T vs C) inheritance models. Our findings did not support an association between AXIN2 rs7224837 and IRF6 rs861019 polymorphism and risk/protection of NSCL/P. The IRF6 2235371 variant was not polymorphic in our population.

**Conclusion:**

The results indicate that the BMP4 rs17563 variant is likely to confer a protective effect against the occurrence of NSCL/P in a sample of the southeast Iranian population.

## Introduction

Nonsyndromic cleft lip with or without cleft palate (NSCL/P) is one of the most common congenital malformations among live births worldwide^5^. The prevalence of NSCL/P is about 1.7 *per* 1000 live births, although this rate is different in terms of geographical position and ethnicity^18^, with higher occurrence in Asian and Native American populations than in African populations. Although the exact etiology of NSCL/P is unknown, it is proposed that both genetic and environmental factors play a role in the disease's pathogenesis^5^.

Wnts are a family of signaling molecules that play critical roles in diverse aspects of craniofacial development. The AXIN2 (axis inhibition protein 2) acts as a tumor suppressor gene in numerous cancers mapped at human chromosome 17q23-q24^6^. AXIN2 serves as a scaffolding component of the multiprotein complex and negatively regulates Wnt/β-catenin, signaling downstream pathway via degradation of β-catenin^2^. It has been shown that members of the Wnts gene family are associated with clefts in humans and mice^11^. Mutations or polymorphisms in AXIN2 have been shown to be associated with increased susceptibility to cancer^9^ ^,^ ^10^ and familial tooth agenesis^10^ ^,^ ^16^.

Bone morphogenetic proteins (BMPs) are secreted signaling molecules that belong to the transforming growth factor-b (TGF-b) superfamily of growth factors. The bone morphogenetic protein 4 (BMP4) gene is located at chromosome 14q22-23, contains four exons, and encodes the BMP4 protein^28^, which play critical roles in embryonic development. Animal models indicated that the BMP4 is a potential candidate gene for NSCL/P^14^. Liu, et al.^13^ (2005) reported that all embryos of BMP4 knockout in a mouse model had bilateral CL at the 12^th^ embryonic day, demonstrating that the BMP signal pathway is critical for cell proliferation and fusion of oral and maxillofacial construction. The rs17563 (538T/C) functional polymorphism of the BMP4 gene is shown to be associated with risk of NSCL/P development ^7^.

The interferon regulatory factor 6 (IRF6) gene is located on chromosome 1q32.2. It has been shown that variants of the IRF6 gene increased the risk of NSCL/P as well as tooth agenesis^20^ ^,^ ^22^ ^,^ ^26^.

Genetic risk factors for NSCL/P may be attributed to ethnic and environmental differences among various populations. Accordingly, repeating previously described genetic associations in different populations is required to define the associations of the genetic risk in each population. Therefore, this study aimed to evaluate the possible association among AXIN2 rs7224837, BMP4 rs17563, and IRF6 rs861019 and 2235371 polymorphisms and NSCL/P risk in a sample of the southeast Iranian population.

## Materials and Methods

A total of 288 subjects, including 132 NSCL/P and 156 healthy subjects, were recruited for this case-control study. The criteria for participants with NSCL/P and healthy individuals were previously described^22^ ^-^ ^24^. The local Ethics Committee of the Zahedan University of Medical Sciences approved the project (IR.ZAUMS.Rec.1393.6923) and informed consent forms were collected from all participants (or their legal guardians). Blood samples were collected in EDTA containing tubes from patients and controls, and then stored at -20°C. Genomic DNA was extracted by salting-out method.

### Genotyping

**Figure 1:**
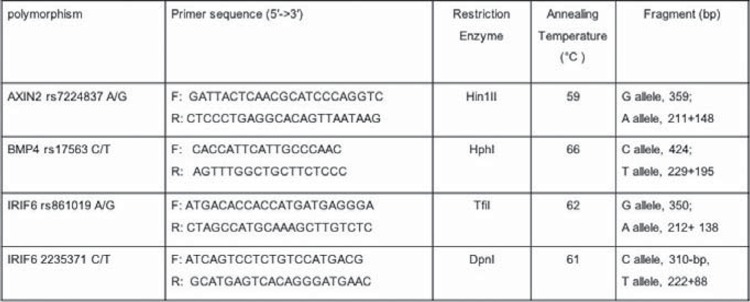
The primers and restriction enzymes used for detection of AXIN2, BMP4, and IRF6 polymorphisms using PCR-RFLP methods

Genotyping of AXIN2, BMP4 and IRF6 variants was carried out using the polymerase chain reaction-restriction fragment length polymorphism (PCR-RFLP) method. The primers sequences and amplicon sizes are shown in Table 1. PCR was performed using commercially available Prime Taq premix (Genetbio, South Korea) according to the manufacturer's recommended protocol. Into each 0.20 ml PCR reaction tube, 1 μl of genomic DNA (~100 ng/ml), 1 μl of each primer (10 μΜ), 10 μl of 2X Prime Taq Premix, and 7 μl of ddH20 were added. PCR cycling conditions were initial denaturation at 95°C for 5 min followed by 30 cycles for 30 s at 95°C, and annealing temperature (Table 1) for 30 s, extension at 72°C for 30 s, with a final extension of 72°C for 10 min. The PCR products (10 μl) were digested by appropriate restriction enzymes (Table 1) and the digested products were electrophoresed on 2.5% agarose gel containing 0.5 μg/mL ethidium bromide and visualized on a UV transilluminator (Figure 1).

**Table 1:** Genotype and allele frequencies of AXIN2, BMP4, and IRF6 polymorphisms in CL/P and control subjects

Polymorphism	NSCL/P	Control Patients	OR (95%CI)	P-value
	n (%)	n (%)		
AXIN2 rs7224837 A>G				
Codominant				
AA	103 (78.0)	125 (80.1)	1	-
AG	25 (19.0)	26 (16.7)	1.17 (0.63-2.14)	0.643
GG	4 (3.0)	5 (3.2)	0.97 (0.25-3.71)	0.973
Dominant				
AA	103 (78.0)	125 (80.1)	1	-
AG+GG	29 (22.0)	31 (19.9)	1.14 (0.64-2.01)	0.665
Reccessive				
AA+AG	128 (97.0)	151 (96.8)	1	
GG	4 (3.0)	5 (3.2)	0.94 (0.25-3.59)	0.944
Overdominant				
AA+GG	107 (81.0)	130 (83.3)	1	-
AG	25 (19.0)	26 (16.7)	1.17 (0.64-2.14)	0.644
Allele				
A	231 (87.5)	276 (88.4)	1	-
G	33 (12.5)	36 (11.6)	1.09 (0.66-1.81)	0.797
BMP4 rs17563				
Codominant				
CC	121 (91.7)	120 (76.9)	1	-
CT	10 (7.6)	27 (17.3)	0.36 (0.17-0.79)	0.012
TT	1 (0.7)	9 (5.8)	0.11 (0.01-0.88)	0.019
Dominant				
CC	121 (91.7)	120 (76.9)	1	-
CT+TT	11 (8.3)	36 (23.1)	0.30 (0.15-0.62)	0.0007
Recessive				
CC+CT	131 (99.3)	147 (94.2)	1	-
TT	1 (0.7)	9 (5.8)	0.12 (0.02-0.99)	0.023
Overdominant				
CC+TT	122 (92.4)	129 (82.7)	1	-
CT	10 (7.6)	27 (17.3)	0.39 (0.18-0.84)	0.021
Allele				
C	252 (95.4)	267 (85.6)	1	-
T	12 (4.6)	45 (14.4)	0.28 (0.15-0.55)	<0.0001
IRF6 rs861019 G>A				
Codominant				
GG	54 (40.9)	50 (32.1)	1	-
GA	63 (47.7)	86 (55.1)	0.68 (0.41-1.12)	0.157
AA	15 (11.4)	20 (12.8)	0.69 (0.32-1.50)	0.435
Dominant				
GG	54 (40.9)	50 (32.1)	1	-
GA+AA	78 (59.1)	106 (67.9)	0.68 (0.42-1.01)	0.139
Recessive				
GG+GA	117 (88.6)	136 (87.2)	1	-
AA	15 (11.4)	20 (12.8)	0.87 (0.43-1.78)	0.722
Overdominant				
GG+AA	69 (52.3)	70 (44.9)	1	-
GA	63 (47.7)	86 (55.1)	0.74 (0.47-1.83)	0.237
Allele				
G	171 (64.8)	186 (59.6)	1	-
A	93 (35.2)	126 (40.4)	0.80 (0.57-1.27)	0.228

### Statistical analysis

All statistical analyses were performed using the SPSS 22.0 software. The associations between alleles or genotypes and NS-CL/P were assessed by computing the odds ratio (OR) and 95% confidence intervals (95%CI) from unconditional logistic regression analyses. P-values less than 0.05 were considered statistically significant.

## Results

This study consisted of 132 NSCL/P (78 males, 54 females, age: 12.13±11.68 years) and 156 healthy individuals (94 males, 62 females, age: 11.35±11.81 years). Of the 132 patients, 52 had CL, 44 had CL with CP (CLP), and 36 had CP. There were no statistically significant differences between the groups regarding sex and age (p=0.904 and p=0.575, respectively). Genotypic and allelic frequencies of AXIN2, BMP4 and IRF6 polymorphisms are shown in Table 2.

**Table 2:** Frequency distribution of genotypes combinations for the IRF6 rs861019, and BMP4 rs17563, and AXIN2 rs7224837polymorphisms between patients with nonsyndromic cleft lip and/or cleft palate (NSCL/P) and control subjects

IRF6 rs861019	BMb4 rs17563	Axin2 rs7224837	Cases	Controls	OR (95%CI)	p
			n (%)	n (%)		
GG	CC	AA	41 (31.1)	35 (22.4)	1	-
GA	CC	AA	44 (33.6)	51 (32.7)	0.74 (0.40-1.35)	0.357
AA	CC	AA	11 (8.3)	10 (6.4)	0.94 (0.36-2.47)	0.939
GG	CT	AA	2 (1.5)	5 (3.2)	0.34 (0.06-1.87)	0.254
GA	CC	AG	12 (9.1)	9 (5.8)	1.14 (0.43-3.02)	0.945
GA	CT	AA	4 (3.0)	15 (9.6)	0.23 (0.07-0.75)	0.013
GG	CC	AG	7 (5.3)	4(2.6)	1.49 (0.40-5.53)	0.747
GG	TT	AA	1 (0.8)	4(2.6)	0.21 (0.02-2.00)	0.19
AA	CC	AG	3 (2.3)	7 (4.5)	0.36 (0.09-1.52)	0.191
GG	CT	AG	1 (0.8)	1 (0.6)	0.85 (0.05-14.16)	0.912
GA	CT	AG	1 (0.8)	3 (1.9)	0.28 (0.03-2.86)	0.341
AA	CT	AG	1 (0.8)	1 (0.6)	0.85 (0.05-14.16)	0.912
GG	CC	GG	2(1.5)	1 (0.6)	1.71 (0.15-19.65)	0.933
GA	CC	GG	1 (0.8)	2 (1.3)	0.42 (0.04-4.91)	0.597
GA	CT	GG	1 (0.8)	1 (0.6)	0.85 (0.05-14.16)	0.912
AA	CC	GG	0 (0.0)	1 (0.6)	-	-
AA	CT	AA	0 (0.0)	1 (0.6)	-	-
GA	TT	AA	0 (0.0)	4(2.6)	-	-
GA	TT	AG	0 (0.0)	1 (0.6)	-	-

The findings revealed that BMP4 rs17563 polymorphism significantly decreased the risk of NSCL/P in codominant (OR=0.36, 95%CI=0.17-0.79, p = 0.012, CT vs CC and OR=0.11, 95%CI = 0.01­ 0.88, p = 0.019, TT vs CC), dominant (OR=0.30, 95%CI=0.15-0.62, p=0.0007, CT+TT vs CC), recessive (OR=0.12, 95%CI = 0.02-0.99, p = 0.023, TT vs CC+CT), and overdominant (OR=0.39, 95%CI=0.18-0.84, p=0.021, CT vs CC+TT) inheritance models. The T allele significantly decreased the risk of NSCL/P (OR=0.28, 95%CI=0.15-0.55, p<0.0001), compared to the C allele.

Our findings did not support an association between AXIN2 rs7224837 and IRF6 rs861019 polymorphisms and risk/protection of CL/P in any inheritance model tested (Table 1). In addition, The IRF6 rs2235371 variant was not polymorphic and all cases (including controls) were CC genotype.

We also analyzed gene-gene interaction (Table 2). In comparison to the reference IRF6 rs861019 GG, BMP4 rs17563 CC, AXIN2 rs7224837 CC, and the GA/CT/AA genotype significantly decreased the risk of NSCL/P (OR=0.23, 95%CI = 0.07-0.75, p=0.013).

We also stratified the patients into cleft lip (CL), cleft lip with cleft palate (CLP), and cleft palate (CP) and assessed the impact of the polymorphisms and the risk of the disease by comparing with the controls (Table 3). The findings showed that the BMP4 rs17563 variant was significantly associated with decrease risk of CL, CLP, and CP. AXIN2 rs7224837 and IRF6 rs861019 were not associated with the risk of CL, CLP, or CP.

**Table 3:** Genotype and allele frequencies of AXIN2 rs7224837, BMP4 rs17563, and IRF6 rs861019 gene polymorphisms in subjects with cleft lip (CL), cleft lip with cleft palate (CLP), and cleft palate (CP)

Polymorphism	Controls	CL	OR (95%CI), p	CLP	OR (95%CI), p2	CP	OR (95%CI), p3
	(n)	(n)		(n)		(n)	
Axin2 rs7224837							
AA	125	41	1	35	1	27	1
AG	26	9	1.06 (0.46-2.44), 0.953	7	0.96 (0.38-2/40), 0.911	9	1.60 (0.67-3.81), 0.341
GG	5	2	1.22 (0.23-6.53), 0.917	2	1.43 (0.27-7.68), 0.651	0	-
A	276	91	1	77	1	63	1
C	36	13	1.09 (0.56-2.16), 0.861	11	1.09 (0.53-2.25), 0.851	9	1.09 (0.50-2.39), 0.839
BMP4 rs17563							
CC	120	48	1	39	1	34	1
CT	27	3	0.28 (0.08-0.96), 0.040	5	0.57 (0.21-1.58), 0.360	2	0.26 (0.06-1.16), 0.074
TT	9	1	0.28 (0.03-2.54), 0.288	0	3.05 (0.06-156.4), 0.983	0	0.18 (0.01-3.24), 0.206
C	267	99	1	83	1	70	1
T	45	5	0.30 (0.12-0.78), 0.008	5	0.36 (0.14-0.93), 0.028	2	0.17 (0.04-0.72), 0.005
IRF6 rs861019							
GG	50	22	1	14	1	18	1
GA	86	25	0.66 (0.34-1.29), 0.231	25	1.04 (0.49-2.18), 0.912	13	0.42 (0.19-0.93), 0.042
AA	20	5	0.57 (0.19-1.71), 0.438	5	0.89 (0.28-2.81), 0.927	5	0.69 (0.23-2.13), 0.598
G	186	69	1	53	1	49	1
A	126	35	0.75 (0.47-1.19), 0.246	35	0.98 (0.60-1.58), 0.965	23	0.69 (0.40-1.19), 0.227

**Figure 2:**
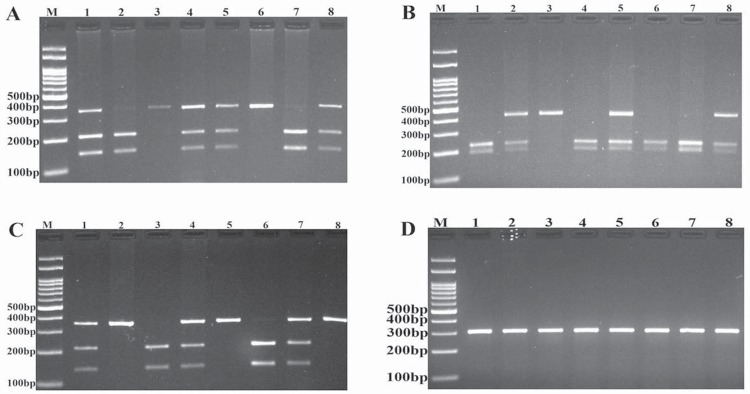
Photograph of electrophoresis pattern of the PCR-RFLP method for detection of AXIN2 rs7224837 (A), BMP4 rs17563 (B), IRF6 rs861019 (C) and IRF6 2235371 (D). M: DNA marker. For AXIN2 rs7224837 variant, lanes 1, 4, 5, and 8: AG; lanes 2, and 7: AA; lanes 3, and 6: AA. For BMP4 rs17563 polymorphism, lanes 1, 4, 6, and 7: TT; lanes 2, 5, and 8: CT; lane 3: CC. For IRF6 rs861019 variant, lanes 1, 4, and 7: GA; lanes 2, 5, and 8: GG; lanes 3, and 6: AA. Regarding IRF6 2235371 variants, all lanes are CC genotype

## Discussion

The documentation of risk factors according to ethnicity and geographic origin is important for understanding the major causes of NSCL/P.

We have previously shown an association between FGF1 rs34010, CDH1 rs16260, MSX1 rs12532 and DHFR 19-bp insertion/deletion polymorphisms and NSCL/P in a sample of the southeast Iranian population^22^ ^-^ ^24^. In this study, we examined the impact of AXIN2, BMP4 and IRF6 polymorphisms on the risk of NSCL/P. The results suggested that BMP4 rs17563 polymorphism significantly decreased the risk of NSCL/P in codominant, dominant, recessive, and allele inheritance models. No significant association was found between AXIN2 rs7224837 and IRF6 rs861019 variants and risk of NSCL/P. We observed that rs2235371 polymorphism of the IRF6 gene was not polymorphic, and all cases (including controls) were CC genotype.

Letra, et al.^12^ (2012) have shown a significant association between AXIN2 rs7224837 variant and risk of NSCL/P in various populations, including Latin America, Europe, and Asia. Mostowska, et al.^19^ (2012) have found that rs3923087 polymorphism was associated with decreased risk of NSCL/P in a Polish population. However, they did not find an association between rs4074947, rs7224837, and rs2240308 variants of AXIN2 and the risk of the disease. A meta-analysis performed by Wang, et al.^30^ (2012) showed that rs2235371 variant significantly decreased the risk of NSCL/P. Recently, Machado, et al.^15^ (2017) showed that the minor A allele of rs7210356 as and the T-G-G-A-G haplotype formed by rs7591, rs7210356, rs4791171, rs11079571, and rs3923087 polymorphisms in the AXIN2 gene were significantly associated with NSCL/P in the Brazilian population.

Mijiti, et al.^17^ (2015) investigated the impact of several polymorphisms of the IRF6 gene on NSCL/P in Xinjiang, China. They observed that the rs7545538 variant significantly increased the risk while rs2235377, rs2235371, 209968684, rs2013162, and rs861019 variants were associated with decreased risk of NSCL/P. The other variants were not associated with risk of NSCL/P.

Several previous studies have shown that BMP4 can be implicated in the development of CL/P. A meta-analysis performed by Hu, et al.^7^ (2015) showed that the BMP4 rs17563 variant could play a different role in NSCL/P according to ethnicity diversity. In the Chinese population, this variant significantly increased the risk of NSCL/P while it presented a protective effect in the Brazilian population^1^ ^,^ ^7^. On the other hand, Chen, et al.^4^ (2012) reported that this variant was not associated with NSCL/P in an Asian population.

The association of IRF6 rs2235371 polymorphism and NSCL/P has been extensively investigated, but the findings are still controversial^3^ ^,^ ^8^ ^,^ ^17^ ^,^ ^20^ ^,^ ^21^ ^,^ ^27^ ^,^ ^29^. Tang, et al.^27^ (2009) reported that the T allele of rs2235371 may confer an increased risk of developing cleft in Chinese individuals. In contrast, another Chinese study has revealed that the rs2235371 variant appears to be protective^17^. In this study, we found that this variant is not polymorphic in our population. It has been reported that 12% of the genetic contributions to CL/P is related to IRF6 variants. The rs2235371 variant of IRF6 was established to increase the rate of recurrence of cleft condition to 9%^33^. Souza, et al.^26^ (2016) reported that the IRF6 G/A haplotype (rs2235371/rs642961) increased the risk for oral cleft in the Brazilian population. A meta-analysis performed by Wattanawong, et al.^31^ (2016) showed that the IRF6 rs2235371 variant decreased the risk of NSCL/P for Asian populations but not Caucasian populations. They reported that the rs2013162 variant decreased the risk of NSCL/P in Caucasian poulations but not in Asian. In addition, the finding suggest that rs642961 and rs987525 variants of IRF6 significantly increase the risk of NSCL/P in both Asian and Caucasian populations^31^. Xu, et al.^32^ (2016) found that rs2235371 and rs2013162 polymorphisms of IRF6 were associated with NSCP in the Chinese population. Recently, Salagovic, et al.^25^ (2017) reported that the IRF6 rs642961 variant significantly increased the risk of NSCL/P in the Slovakian population.

The inconsistent results may arise from different ethnic origins, environmental differences, and the complex genetic etiology of the NSCL/P disease.

One of the limitations of this study is its relatively small sample sizes. Another limitation is that we evaluated limited variants of the AXIN2, BMP4, and IRF6. Other genetic variants of these genes should also be evaluated.

In conclusion, we performed an association study of AXIN2, BMP4 and IRF6 gene SNPs with NSCL/P in an Iranian population and the findings proposed that BMP4 rs17563 polymorphism is associated with reduced risk against NSCL/P. Future studies with larger samples from different ethnicities are needed to confirm our findings.
